# Measurement of Food Losses in a Hungarian Dairy Processing Plant

**DOI:** 10.3390/foods10020229

**Published:** 2021-01-23

**Authors:** Katalin Tóth, Csaba Borbély, Bernadett Nagy, Gábor Szabó-Szentgróti, Eszter Szabó-Szentgróti

**Affiliations:** 1 Institute of Regional and Agricultural Economics, Szent István University Kaposvár Campus, H-7400 Kaposvár, Hungary; borbely.csaba@szie.hu; 2 Institute of Methodology, Szent István University Kaposvár Campus, H-7400 Kaposvár, Hungary; nagy.bernadett@szie.hu; 3 Institute of Marketing and Management, Szent István University Kaposvár Campus, H-7400 Kaposvár, Hungary; szabo-szentgroti.gabor@szie.hu (G.S.-S.); szabo-szentgroti.eszter@szie.hu (E.S.-S.)

**Keywords:** food loss, milk products, dairy industry, FLW standard, measurement

## Abstract

The phenomenon of food waste and food loss at any stage of the supply chain is significant in developed economies. The purpose of this article is to highlight the areas of milk processing where milk loss occurs, and, after quantifying the data obtained, reveal the extent of the losses. To achieve the goals, we conducted on-site visits to one of Hungary’s milk processors. The methodology is based on the Food Loss and Waste (FLW) standard, accordingly we determined the extent of milk loss at the company level, supplemented with loss values by each dairy product. During the analyzed processing stages (receiving of raw milk, skimming, pasteurization, Extended Shelf-Life (ESL) milk, cheese milk, sour cream, yoghurt, and kefir) 1203.4–1406.8 L of raw material per day can be accounted as losses, which makes up 0.9–1% of daily production. A Milk Production-Milk Losses (MPML) model was created where six factors (technology and automation, design of the plant aspects, quantity of orders, expertise of employees, number of product variants, optimal storage capacity) were methodized that significantly influence the rate of milk losses over different time periods. Our paper highlights how areas of the production stage can be developed to decrease milk loss.

## 1. Introduction

While one in eight people worldwide is starving or is underfed [1], in the USA, because of cheap, easily available food and extensive marketing activity, the inhabitants have difficulty adjusting their need for nutrition with their intake, which is the reason for the increasing number of obese people year after year, and the level of food-waste is also rising [2]. One of the main objectives of the Sustainable Development Goals (SDGs) framework signed by 193 countries in 2015 is to reduce food-waste (Goal 2), which contributes to decreasing the level of starvation, or even end it by 2030 [3]. 

Food loss, besides the consumption of resources (land, water, energy, input materials) increases the emission level of greenhouse gases [1]; at the same time there are existing business models and aspirations which focus on reducing food waste from farms, wholesale, the catering industry and consumers in a way that encourages the production of energy from waste [4]. According to the World Food and Agriculture Organization (FAO) [1], vegetables, fruits, roots, and tubers end up in trash cans due to their perishability. By examining each stage of the food chain separately, we can count losses in the production phase due to pests, diseases, or weather, and expect products to be damaged during production or storage, along with storage loss due to non-convenience of quality or size. However, it is worth noting that weather risks not only affect production and product quality, but also the operation of the entire supply chain, which can also lead to losses [5,6]. In the trade phase, inadequate stock management, over-ordering, and limited access to food redistribution systems lead to food waste, but changes in consumer tastes and preferences can also be a reason for loss. In the case of final consumption, the confusion between expiry date and shelf life, overbuying and limited knowledge result in waste for consumers, while in catering inadequate stock management, handling, storage, and barriers to donation are the causes of loss [7].

In the European Union fish and fish products (51%) have the highest ratio of food waste as a proportion of total quantity produced. This is followed by vegetables (46%) and fruits (41%). For milk and milk products, this value is around 5% in total, of which households (62%) and processing (16%) are the most wasteful from the perspective of the supply chain stages [8].

Based on a European Commission [9] study, the main causes of food waste during processing can be traced back to deficiencies in packaging, logistical disruptions, quality requirements for products, and technical defects. A study by Darvasné Ördög et al. [7] refers to German authors who suggest that the main cause of loss in the manufacturing industry is product defect, technical defect, and expiration of warranty. In contrast, in their research, technologically avoidable factors (e.g., spillage, scatter, manufacturing error, poor labelling), human fault and technologically unavoidable factors were identified as the most common reasons for food loss. Unavoidable technological factors were given the highest value according to their importance. By no means can we say that the references in the literature are the same as the causes of losses in the food industry. The ranking of causes is also very variable in the literature. In our opinion, this may be due to the level of development of the examined country/region, the examined year, the range of sub-sectors involved in the research, and technological differences among the companies.

**Figure 1:**
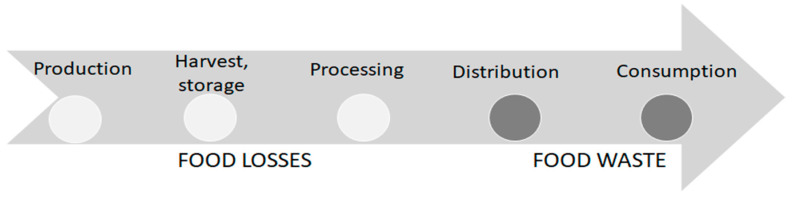
Food loss and waste according to their position in the supply chain. Source: based on Parfitt et al. [11]; Food and Agriculture Organization (FAO) [10], own editing.

Food loss is nothing more than a reduction in the weight or quality of food in the food supply chain. According to one approach, depending on which stage of the production chain is affected by the decline, the literature defines it as food loss or food waste [10] (Figure 1). At the beginning of the supply chain (production, harvesting, processing) food losses are caused by logistical and infrastructural barriers, while at the end (retail, consumer) prodigality of food generates waste, and the latter also reflects a kind of consumer behaviour [11]. In our case, we use the concept of food loss, because our research is limited to manufacturing industry.

In another approach, “food loss is the amount of food that is produced for human consumption but gets out of the supply chain for different reasons. Food waste is a subset of food loss and represents the amount of food, still suitable for consumption, which is discarded as a result of human action or inaction” [12] (p. 12).

One important finding of Östergren et al. [13] is that different definitions are used for the same terms in studies and reports on food waste. In some studies, loss is measured in mass, while other authors account for it in money. If the nutritional value of each food is also taken into account, the difference can be further increased. According to Directive 2008/98/EC on waste (Waste Framework Directive), which is often used in the manufacturing industry, waste is “any substance or object which the holder discards or is required to discard” [13] (p. 44). Based on this, the question rightly arises: is the by-product counted as waste? For example, by-products from the food and beverage industry used for animal feed do not fall within the legal definition of “waste”.

Due to earlier mentioned methodological differences, the measurement results of records may also vary on the same topic. There is also no consensus at the level of the various disciplines on which sector is the most or least wasteful [14]. Thus, we clearly cannot determine where the dairy industry is ranked based on food loss, which does not call into question the importance of the topic, but only confirms that it is still relatively new and more research is underway at this time. 

A study by Themen [15] examining losses in different food product chains (cereals, meat and meat products, fruit and vegetables, dairy products, etc.) drew general conclusions about the losses “suffered” by each country along product lines based on their income situation. In the study, Hungary, as a member of the EU-27, is classified as a high-income country, so the usual findings are true, i.e., in the case of dairy products, the losses and wastes generated during the consumption phase are the highest, in total 7% of purchased products. Losses in the production phase are at around 5%; meanwhile the same around 20% in middle-income countries due to poor milking practices and husbandry technology. This loss rate also affects the liquidity and profitability of agriculture, of which the overall situation in Hungary is analysed by Varga & Sipiczki [16]. During the refrigeration, storage and distribution of dairy products, at least 0.6% losses can be expected. Processing causes around 1.3% of losses, although in proportion most losses are incurred in low-income countries because of hygiene conditions and technology [15].

According to a study covering four dairy processing farms in the UK (25% of total production), the most significant loss resulted from “separator de-sludge”, which is a consequence of the separation process between cream and milk. The proportion of this amount of milk loss is approximately 0.2% of the intake. Other milk losses occur in milk processing plants during cleaning, line changes, and product rejection, but cleaning/switchover losses directly attributable to milk were not determined in that study [17].

Nestlé applies loss reduction at all stages of its supply chain based on the FLW standard. To quantify losses, process mapping is used first, followed by observations and interviews to estimate the loss rate, which confirms that the total milk loss and waste is 1.4% of processed milk along the whole value chain, where 40% is generated by farmers [18,19]. However, we did not find a numerical estimate for Nestlé that would reveal the extent of losses for each product, nor does the cited study separate the treatment of losses (destination) for each stage of the supply chain. 

As in previous surveys [7,13], waste generated during production—if recycled e.g., in the form of cheese spread or milk drink—are not considered as losses.

Among the milk processing waste reduction options [17]—longer shelf life, freezing, utilizing separator de-sludge—we do not currently consider any of them feasible. The most pressing of the three issues is to utilize separator de-sludge, since according to Ahmad et al. [20], the dairy industry, in addition to having high water consumption, allows large amounts of dairy products to enter the drain. Sewage has a high organic matter content, biological and chemical oxygen demand, and temperature. If it is not handled properly and placed directly into the soil, it can cause serious environmental problems and harm people, aquatic organisms, and agriculture. 

Measuring milk product chain losses has been the subject of several articles and studies [18,19,21,22,23]. Carawan & Jones [21] dealt with the management of losses in the dairy industry in the late 1970s; although the technology of that time is now obsolete, it is worth paying attention to some of the authors’ findings that are still valid today. According to the authors, human resource expertise, management training on the subject and the role of the specialist responsible for the treatment of the wash water (hereinafter referred as ‘water’) are key in reducing losses. They also stress the importance of financial savings. Wesana et al. [23] applied the method of value stream mapping (VSM) in their studies. The essence of this method is to visualize the value creation process, thus making each step of the process visual and measurable so it will be possible to see where the losses arise in the process. This was based on the FLW standard developed by Hanson et al. [24,25], and Charad et al. [18], Tostivint et al. [19], and Charad et al. [22] utilized this as the base for their methods; however, the information on milk losses is somewhat approximate, and a level of data that would determine the extent of milk losses for each product cannot be found.

A review of the literature has made it clear that there are currently no examples to follow to determine the possible causes and extent of milk loss at a certain product level. This research seeks to fill this gap. The purpose of this article is to highlight the areas of milk processing where milk loss occurs, quantifying the data obtained. Based on this, the following research questions were formulated:

1. Is it possible to quantify, and if so, to what extent (in milk equivalent) the amount of food loss in milk processing?
2. At which production stage does the greatest amount of food loss in milk processing occur?
3. How can we reduce the amount of food loss in milk processing?

## 2. Materials and Methods 

**Figure 2:**
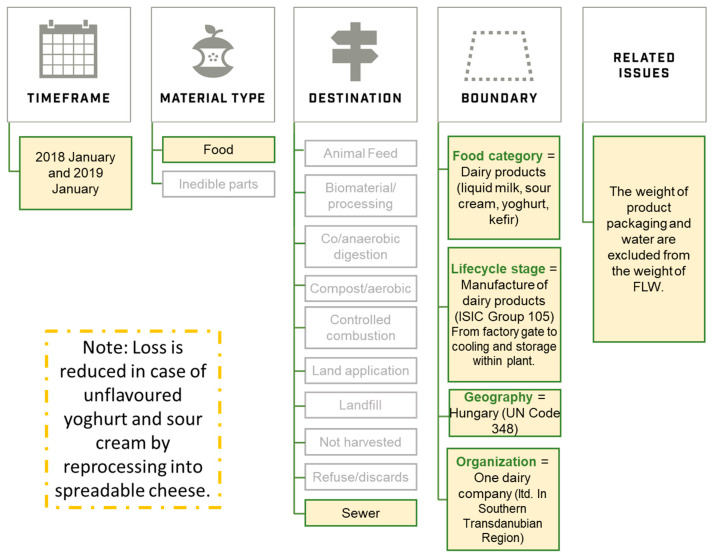
The investigated company’s framework of loss based on the FLW standard.

The methodology of the present study is based on the FLW standard (Figure 2), and accordingly, we determined the extent of milk loss at a company level, supplemented with loss values by each dairy product. Hereinafter, loss means the loss of raw milk or dairy products.

### Flw Standard Requirements & Description of the Analysed Company’s Inventory

1. Base FLW accounting and reporting on the principles of relevance, completeness, consistency, transparency, and accuracy.
  ▹ Relevance: Milk processing was evaluated to identify relevant hotspots, where milk and dairy product losses and waste were higher than expected and therefore could be reduced.
  ▹ Completeness: From factory gate (intake of raw milk from transport vehicle) to cooling and storage within the plant.
  ▹ Consistency: Two on-site visits combined with expert interviews. Data was ensured by production management.
  ▹ Transparency: In the case of liquid milk, yoghurt, sour cream, and kefir, destinations and quantity of losses were reported transparently by experts. In the case of cheese products, no access was allowed. The name of the analysed dairy company is withheld at the request of the management.
  ▹ Accuracy: depends on how the measurement is performed (Table 1). Liquid milk product (ESL) loss estimations are more accurate than for yoghurt, sour cream, and kefir.
2. Account for and report the physical amount of FLW expressed as weight.

Reported as kilogram per day. Average loss amount in milk equivalent (kg/day). In the case of liquid milk, the milk equivalent was calculated based on values set by the European Commission [26]; in the case of plastic cup dairy products—as allowed by the European Commission’s regulation—the experts provided milk equivalent data. Products, which are generated during processing and are recycled in a later process are not considered to be losses.

**Figure 3:**
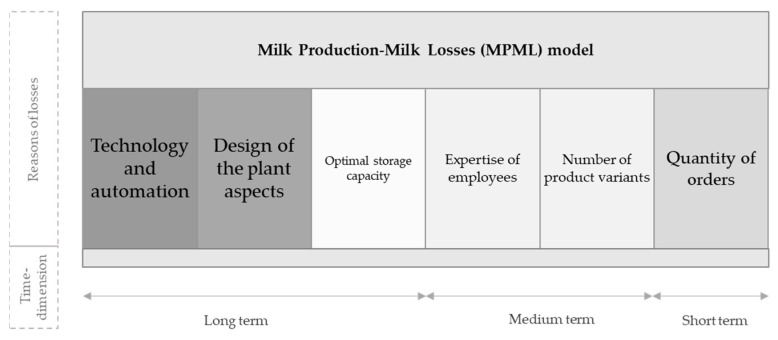
Milk Production-Milk Losses (MPML) model. Note: Causes occurring in most phases were marked with larger font size and a darker background. As the number of occurrences decreases, the font size decreases and the background colour becomes lighter.

3. Define and report on the scope of the FLW inventory (see FLW Standard for additional details).
  Timeframe: Data reported for January 2018 and January 2019.
  Material type: Food (inedible parts were not included)
  Destinations: When water is mixed with milk, sewer/wastewater treatment was considered as milk loss and waste. Clean water or other losses were not currently the subject of our research.
  Boundary:
  ▹ Food category: dairy products (General Standard for Food Additives (GSFA) 01.0): liquid milk, sour cream, yoghurt, kefir.
  ▹ Lifecycle stage: From factory gate (intake of raw milk from transport vehicle) to cooling and storage within the plant (ISIC code: 1050—manufacture of dairy products)
  ▹ Geography: Hungary, UN country code 348
  ▹ Organization: One Southern Trans-Danubian dairy factory (ltd).
  Related issues: Weight of packaging and water are excluded.
4. Describe the quantification method(s) used. If existing studies or data are used, identify the source and scope.
  Surveys (visits and interviews):
  ▹ January 2018: identification of processing stages and loss data was ensured (Table 2). Whole plant visit and senior and junior production manager interviews.
  ▹ January 2019: revision of loss data from January 2018 (no significant change was reported). Partial plant visit and senior and junior production manager interviews.
  ▹ March 2020: finalization of identified reasons of milk loss (Figure 3). Senior production manager online interview.
5. If sampling and scaling of data are undertaken, describe the approach and calculation used, as well as the period of time over which sample data are collected (including start and end dates)

Measurement of milk loss is done by the company regularly (Table 1). A detailed time-series database was withheld by the company; received data is structured in Table 2. The amount of total losses is the sum of the losses measured at each processing stage. The extent of the intervals indicates the varying number of systems booting.

6. Provide a qualitative description and/or quantitative assessment of the uncertainty around FLW inventory results.

As FLW data were defined by experts, errors may occur. A digital flow meter gives more accurate data; nevertheless, measuring during cleaning, under pressure with a digital scale, may be led to estimations due to water content.

7. If assurance of the FLW inventory is undertaken (which may include peer review, verification, validation, quality assurance, quality control, and audit), create an assurance statement.

Not applicable.

8. If tracking the amount of FLW and/or setting an FLW reduction target, select a base year, identify the scope of the target, and recalculate the base year FLW inventory when necessary.

Not applicable.

Source: FLW Standard [27].

## 3. Results and Discussion

The results of the empirical research were established based on expert interviews and on-site visits. The technological level of the examined plant—according to the experts working there—represents a medium or slightly higher technological level within Hungary. Improvements are continuous, but full automation is not present in any of the processes. This is also supported by Rózsa & Tálas [28], who examined the financial position of the leading dairy processing companies in Hungary and found that technological development is low, as investment and development loans do not appear in the agencies’ financing. As the extent of the losses depends on the equipment used in the milk processing plant, it was important for us to include these parameters too (Table 1). The average age of the equipment used was 7.7 year at the time of data collection.

The specific quantification of losses in the dairy industry took place along the processing stages and will be described accordingly in our publication. Appendix A shows the production chain that served as the basis for this study.

140,000 L of raw milk are processed daily at this plant, which is examined before intake. The quality of the milk is examined, inter alia, for its physical and microbiological purity. Non-compliant milk is not processed, as only extra quality milk can be. Raw milk rejection happened only once during the investigation period, when 18,000 L of milk were refused, corresponding to a loss of 0.035% per annum. This kind of loss is borne by the farmer, therefore this loss is not included in our analysis.

The first loss arises with the intake of raw milk, which cannot be avoided with the current technology, because this sub-process is fully automated. Since no water can remain in the pipes and silos, some of the milk will inevitably end up in the drain mixed with the water. The tanks are leached twice a day, and for one wash 40–50 L of milk loss can be expected (Table 2). 

Thereafter, loss occurs in the form of separator de-sludge during skimming. According to Babcsányi et al. [29], this means a loss of 0.1–0.2% of the applied material. In the case of the examined company, it is approximately 0.27% i.e., 378 L in milk equivalent, which corresponds to 389 kg from a daily intake of 140,000 L of raw milk. The extent of separator de-sludge depends on the level of technological development, and is an unavoidable loss in the short term. However, in the long run, the amount of saved milk through technological improvements would fall into the category of avoidable losses, which could mean up to 0.17% savings. Separator de-sludge appears as an opportunity to be utilized as a feed with protein content of 3%, but in Hungary dairy industry waste intended for feed usage is under strict regulations. Only properly treated (heat treated, packaged, shipped) material can be used for this purpose, which increases the cost compared to a simple soy protein. The consistency of the material can also be mentioned as an obstacle, as the distribution of liquid feed requires special technology, and this is not typical. 

In the case of the investigated company, the pasteurization process is only partially automated. The pasteurization machine must be started (rebooting) several times a day, on average three times a day. The loss at each start of cleaning under pressure is 30–40 L (Table 2), i.e., we need to count this value six times a day. Only part of this could be avoided if the system were fully automated. In the present case, the milk in the pipes is inspected visually. The earlier mentioned 30–40 L loss, before the current technology, was approximately 80–100 L, so we see a reduction of about 300–360 L per day compared to the previous condition. 

After pasteurization, the milk is used for making ESL dairy products and cheese milk, which is used in turn for making cheese. Cream is used during the making of plastic cup dairy products. During the production of ESL milk, cleaning the pipes results in a maximum of 50 L/occasion loss on average four times a day. Counted in milk equivalent, this is 126 L per day, i.e., 130 kg, which has a much lower milk equivalent (74%) due to the variable fat content, but since it is an under pressure cleaned quantity, at least 15% of it is water, namely 63% of the original quantity. ESL milk is transferred from the buffer tank to the package, where loss does not usually occur. In the case of cheese milk, a loss of 50–60 L/occasion can be expected 6–7 times a day, which is 87.5% of the milk equivalent, since it is lower fat milk.

The loss of plastic cup dairy products is more difficult to calculate, which is made even more difficult by the fact that the production lines operate depending on the order quantity. For example, in the case of yoghurt several flavours are produced on one production line, which is a loss-increasing factor. For plastic cup products, the biggest factor to increase the loss is product switchover (e.g., to a different flavour of yoghurt or from yoghurt to sour cream), hence the most unfavourable situation is if the product remains in the tube. For sour cream, the average loss during cleaning under pressure would be 240–300 kg/week; however, a loss-reducing factor is that the product is recycled in the spreadable cheese department. Consequently, in the case of sour cream, the weekly loss is a maximum of 20% (48–60 kg), with a milk equivalent of 256% (122.9–153.6 L/week). For these products, we can speak about periodic production, which on average means a loss of 24.6–30.7 L of milk per day in milk equivalent. In the case of yoghurt, the situation is similar, but here a slightly higher loss rate (30%) per switchover is counted as loss due to the recycling of 70% of the original material into spreadable cheese products. Hence 12.3–24.6 L per day can be expected in milk equivalent. In yoghurt production only the white product can be recycled, but for flavoured yoghurt this possibility does not exist. In the case of kefir, the loss due to switchover is considered to be minimal. Production takes place three times a week on average, and two switches are required during one production. For plastic cup products, the pipes should be washed every 12 h, even if the variant in taste or product is the same. This period is shorter than 12 h in most cases, which is why the pipes are cleaned more often.

In the case of changing packaging materials, we cannot talk about product loss, but only loss of time. Loading failure outflow no longer occurs with this technology. Sampling as loss for quality control covers a rather small amount, thus we did not take it into account.

The average loss of products (milk, sour cream, yoghurt, kefir) waiting for delivery is 0.1%. These products are scrapped and destroyed at the processor. The legal regulations are very strict about giving them to animals as feed, therefore it is, unfortunately, cheaper to destroy the products. For the time being, Food Banks are not an option to take over these products, because damaged products are more perishable. At the same time the retail sector use this option more often in the case of undamaged products, but this was not the subject of our research.

Based on the information obtained, 1203.4–1406.8 L of milk is lost during processing on average per day, which is 0.9–1% of the daily milk intake (Table 2).

Collecting the causes and sources of losses in the dairy industry, we created the Milk Production-Milk Losses (MPML) model, which is shown in Figure 3. 

In the model, we collected the factors that significantly influence the rate of milk losses over different time periods. By considering the included factors in the model, the extent of milk loss and the range of loss reduction options can be determined more precisely in the future, as it also considers the technological aspects which precede the production process. 

During designing processes, the level of technology used is a loss-reducing factor. The loss of fully closed and automated systems is minimal, because in this case, only unavoidable losses occur.

The more outdated technology a company works with, the greater the amount of avoidable losses. The level of technology may also differ within a plant, as the production line of one product is fully automated, but the production line of another product in the same plant may be more obsolete or completely obsolete technology.

The extent of losses also depends on whether considerations of loss minimization have been considered during the design of the plant. The length and slope of the pipes play a key role in reducing the rate of loss. The cleaning process of the pipes appears several times during the processing of the products, and for this purpose water and nitrogen are used. Nitrogen is used to completely remove water from the pipes, thus the water does not mix with the product. By rationalizing the length and slope of the pipes, the company can save not only raw materials but also water and nitrogen.

According to experts, one of the most significant loss-producing factors is the intermittent arrival of raw milk, and as a consequence the pace of processing is not continuous. This causes losses due to cleaning during reboots, as machines must be restarted several times a day during pasteurization. If all the raw milk entered the plant at the same time corresponding to the production capacity of the plant, the associated losses could be minimized. The number of outages and restarts could be reduced by creating optimal storage capacities. During the interviews, it was stated that the intermittent arrival of raw milk is a common phenomenon in dairies. An additional solution to this could be the optimal scheduling of deliveries, which is only partly the responsibility of the processor. 

In the case of product switchover (e.g., from milk with a fat content of 1.5% to milk with a fat content of 2.8%), the fat content of the pipes and containers can be determined mechanically or in an automated way. The fully automated system delivers the milk as needed, with exactly the right amount of fat percentage, into the system. Mechanically there may be an incorrect fat content adjustment, which is not quantitative but a monetary loss. If the system is not fully automated, a specialist will measure the fat content. In this regard, professional experience is an important factor, as an apprentice worker may incorrectly set a higher fat percentage than he or she needs to.

Each product switchover (for reasons of taste or fat content) results in some loss. The more the number of conversions, the higher the loss rate. The number of product variants therefore influences the level of loss. The fact that each product variant has a separate production line, and no transitions are present, is not a realistic assumption, according to the plant’s experts. 

Consequently, the number of orders is also relevant in quantifying losses. The more fragmented the number of orders is, the more times are needed to switch. The company determines the size of order it produces for products of one flavour or products with a particular fat percentage at any time. In this case, on the one hand, economies of scale and, on the other hand, market considerations are decisive as to whether it is worth fulfilling an order or not. By market consideration we mean, that in some cases an order must be fulfilled in a way that overrides aspects of economies of scale to avoid possible market loss.

Comparing with secondary data [7], the most common cause in the food industry does not truly occur in milk processing (spillage, manufacturing defect, poor labelling), but rather an unavoidable technological cause is typical. This is also due to the fact that the loss appears to be mixed with water, which makes it difficult to measure.

It is not necessarily useful to establish a uniform method for measuring losses, as there will be unique factors for each plant, which are unique fundamentally determine the extent of milk loss. These factors should be considered before measuring or estimating losses. Quantification along the supply chain used in the literature [7,18,19,22,23,30] does not consider the influencing factors of long-term pre-real-time production, which can fundamentally determine the evolution of the level of losses (Figure 3). Based on this, it is advisable to calculate the occurrence of losses right from the design of the plant (slope of pipes, expertise, etc.). 

## 4. Conclusions

In our investigations, it became clear that the amount of losses can be relatively standard or fully variable depending on the product processes. In the case of the examined company, the loss incurred during the pasteurization process is mostly constant, as the consumer demand for liquid milk is almost consistent. For plastic cup dairy products this cannot be said, because the extent of the loss depends on the production design. In the case of plastic cup dairy products, the number of product variants is higher, and the number of orders fluctuates more than in the case of liquid milk. According to Themen [15], processing causes 1.3% losses on average depending on the development of the country. Our results show 0.9–1% milk processing loss which includes 0.27% separator de-sludge loss [17] when 140,000 L of raw milk are processed daily.

The answer to our question of whether the rate of food loss in milk processing can be measured is clearly positive. However, it is also clear that the data obtained cannot be generalized due to the diversity of technological standards. In this regard, experts’ opinions obtained during the preparation of the study have also been confirmed. 

The possibility of reducing food losses in dairy processing can be summarized by the following points based on the Milk Production-Milk Losses (MPML) model (in order of processing stage frequency):

- use the highest possible level of automation (A)
- remove water from the pipes with nitrogen (A)
- rationalization of length and slope of pipes (B)
- fulfilment of order quantity that meets the criteria of the company’s economies of scale (F)
- employ a skilled workforce in manufacturing and keep fluctuation as low as possible (D)
- optimization of production also in case of taste transitions (E)
- development of storage capacity adapted to daily production capacity (C)

The quantification of each factor in the MPML model (assignment of a scoring system, development of a regression model) concerns a further stage of the research.

Besides MPML factors, a loss-reducing option is to recycle the product into spreadable dairy product form, if the company’s product portfolio allows this. Utilization as animal feed will also allow a significant loss reduction. However this will require additional usage of resources (because of strict safety regulations), thus the overall sustainability and loss reduction factor is questionable. Nonetheless, it is used by many companies (e.g., Nestlé), while sustainability goals are followed. 

No previous literature has dealt with the measurement of product-level milk loss [15,17,18,19,20,21,22,23]. This research found that it is possible to measure milk losses at individual product levels but, in addition to real-time production processes, other factors are worth considering—as Verma et al. [31] also acknowledged—to minimize losses, thus contributing to the achievement of sustainability goals. 

The results we have obtained draw the attention of market participants in different frames as to what are the most influencing factors in milk loss during milk product processing and how these factors influence the amount of milk loss. Our paper highlights how areas in the production stage can be developed to decrease milk loss, and contributes to strategy development for technology investments in dairy plant. 

## Figures and Tables

**Table 1:** Technological parameters of the equipment at stages of processing in the analysed plant.

Processing Stage	Applied Technology	Technological Parameters of Equipment	How to Measure Losses?
Intake of raw milk	Milk collection in a closed system	1 line 30,000 L/h (pump, plate refrigerator/cooler) 180,000 L of raw milk storage capacity	Digital flow meter
Skimming (separator de-sludge)	“Self-emptying” skimmer, automatic fat adjuster	1 pc 20,000 L/h	Proportionality of operating time and loss per emptying (periodic measurement)
Pasteurization	Closed system, operation in accordance with the applicable regulations (automatic control and registration of temperature and pressure values)	1 pc 20,000 L/h 1 pc 6000 L/h 1 pc 5000 L/h	Digital flow meters, automatic data recording
Extended Shelf-Life (ESL) sterile container	Closed system, overpressure (2 bar)	15,000 L capacity	Digital flow meters, automatic data recording
Cheese milk	Automatic fat, manual protein adjustment	Using the available 20,000 L/h pasteurizer and separator equipment	Digital flow meters, automatic data recording. Calculation according to standard from the finished product.
Sour cream, Yoghurt, Kefir	Closed system product line	Using the available 20,000 L/h pasteurizer and separator, the 5000 and 6000 L/h pasteurizer, and the homogenizers set up with them	Digital flow meters, automatic data recording, stocking of the finished product. Measuring after cleaning under pressure with a digital scale
Products awaiting delivery (Cooling, storage)	Manual and mechanical handling, storage according to First In First Out (FIFO) inventory valuation	Hand and machine forklifts	Measuring and documenting the difference between products taken over from production and sold.

**Table 2:** Amount of and reasons for dairy losses at each stage of processing.

	Processing Stage	Average Loss Amount		Main Reason of Milk Loss
		Liter, kg/day	Milk Equivalent (kg/day)	
Daily production	Intake of raw milk	80–100 L	82.4–103 kg	A,B,C
	Skimming (separator de-sludge)	-	378 L = 389.3 kg	A,B
	Pasteurization	180–240 L	185.4–247.2 kg	A,D
	ESL sterile container	200 L	126 L = 129.8 kg	A,B,F
	Cheese milk	300–420 L	262.5–367.5 L = 270.3–378.5 kg	A,B,F
Periodic production	Sour cream	9.6–12 kg	24.6–30.7 L = 25.3–31.6 kg	A,B,E,F
	Yoghurt	4.8–9.6 kg	12.3–24.6 L = 12.7–25.3 kg	A,B,E,F
	Kefir	negligible	negligible	A,B,F
Products awaiting delivery, storage	milk, sour cream, yoghurt, kefir	140 L	144.18 kg	D
Total			1203.4–1406.8 L 1239.4–1448.9 kg (Daily 0.9–1.0%)	

**Note**: The main reasons for milk loss are indicated by the following numbers: A—Technology and automation, B—Design of the plant aspects, C—Optimal storage capacity, D—Expertise of employees, E—Number of product variant, F—Quantity of orders.

**Figure A1:**
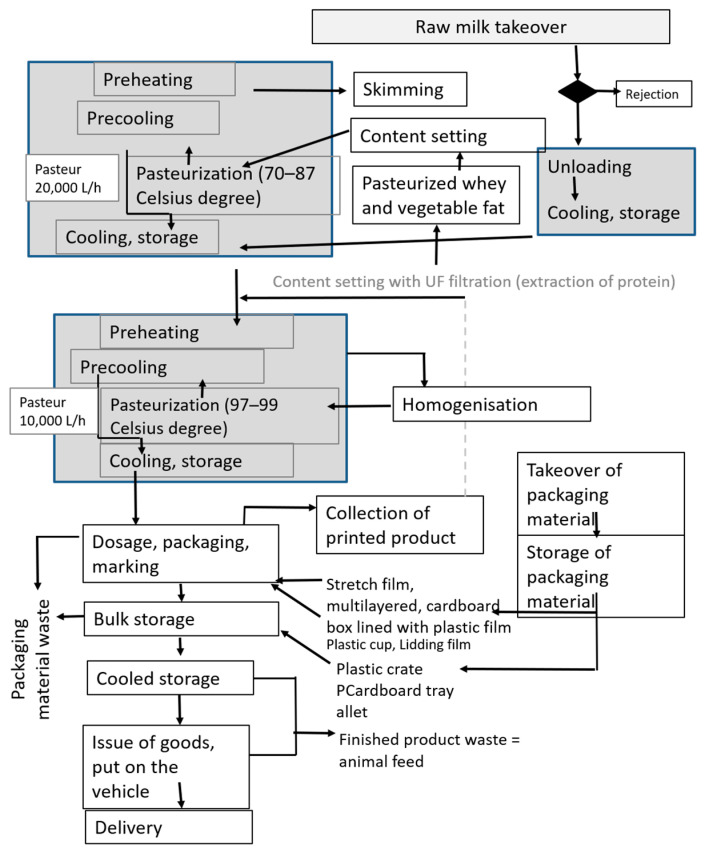
The process of dairy production in the case of the investigated enterprise. Source: Own editing based on the surveyed company data.

## Data Availability

Restrictions apply to the availability of these data. Data was obtained from a Hungarian Southern Trans-Danubian dairy factory and are available with the permission of the company.
